# Metacognitive Awareness of Older Adult Drivers with Mild Cognitive Impairment: Relationships with Demographics, Subjective Evaluation of Cognition, and Driving Self-Efficacy

**DOI:** 10.3390/bs14060483

**Published:** 2024-06-06

**Authors:** Anastasia Tsouvala, Ioanna-Giannoula Katsouri, Despina Moraitou, Georgia Papantoniou, Maria Sofologi, Alexandrina Nikova, Pinelopi Vlotinou, Anna Tsiakiri, Magdalini Tsolaki

**Affiliations:** 1 School of Psychology, Aristotle University of Thessaloniki, 54124 Thessaloniki, Greece; tsouvalaanastasia@gmail.com; 2 Occupational Therapy Faculty, University of West Attica, 12243 Athens, Greece; ykatsouri@uniwa.gr; 3 Laboratory of Psychology, School of Cognition, Brain and Behavior, School of Psychology, Aristotle University of Thessaloniki (AUTh), 54124 Thessaloniki, Greece; demorait@psy.auth.gr (D.M.); tsolakim@auth.gr (M.T.); 4 Lab of Neurodegenerative Diseases, Center for Interdisciplinary Research and Innovation, Aristotle University of Thessaloniki (CIRI—AUTh), 54124 Thessaloniki, Greece; 5 Laboratory of Psychology, Department of Early Childhood Education, School of Education, University of Ioannina, 45110 Ioannina, Greece; gpapanto@uoi.gr (G.P.); m.sofologi@uoi.gr (M.S.); 6 Asclipieio Voulas Hospital, 16673 Voula, Greece; 7 Department of Occupational Therapy, University of West Attica, 12243 Athens, Greece; pakivlot@yahoo.com; 8 Neurology Department, Democritus University of Thrace, 67100 Xanthi, Greece; anniw_3@hotmail.com

**Keywords:** mild cognitive impairment, metacognitive experiences, self-regulation, driving

## Abstract

(1) Self-regulation of driving is a means of maintaining one’s driving identity. The purpose of this study was to investigate the extent to which older drivers with Mild Cognitive Impairment (MCI) are metacognitively aware of the requirements of specific demanding driving conditions and whether this awareness is linked to subjective assessments of cognition. (2) One hundred seventeen (117) older MCI drivers participated in a telephone survey in which they reported their metacognitive experiences in nine driving conditions, listed as an aim of self-regulation. The analyses included the participants’ subjective cognitive assessments, both in terms of their cognitive state and their perceived driving self-efficacy. (3) The analyses pointed out a direct and negative effect of age on the formation of the metacognitive feeling of certainty. Furthermore, an indirect effect of sex through driving self-efficacy was established. This effect was negative in the case of the metacognitive feeling of difficulty and the estimation of effort and positive in the case of the metacognitive feeling of certainty. (4) This position points out the need to establish appropriate levels of the perceived self-efficacy of older drivers with MCI, and it raises issues when it moves to fictitious levels.

## 1. Introduction

According to the American Occupational Therapy Association [1], driving is classified among the Instrumental Activities of Daily Living (IADLs), providing individuals with the ability to live and function independently. Driving requires the simultaneous activation of motor, sensory, perceptual, and mental functions, making it a useful index of an individual’s mental capacity [2,3]. Considering the negative association between age and safe driving, the increase in older drivers brings to the fore the heightened risk of traffic accidents among physically vulnerable older adults [4,5,6,7]. Driving errors, such as misadjusting the speed and distance from the vehicle in front, dangerous overtaking, and daydreaming, are cited as the main reasons for potential crashes [8,9,10]. In this study, the relationships between the subjective evaluation of the cognitive state of individuals regarding the cognitive failures they acknowledge on a daily basis, their driving self-efficacy, whether they feel capable of successfully executing the driving task, and their metacognitive experiences related to the driving task, will be examined. The effects of sex, age, and education will also be analyzed. The population of interest consists of older drivers with Mild Cognitive Impairment (MCI).

Mild Cognitive Impairment (MCI) refers to the appearance of deficits in one or more cognitive areas without a concomitant decline in daily functioning, representing a transitional stage between healthy aging and dementia, with an annual progression rate of 10–15% [11]. However, even at this stage, deficits in driving parameters can be observed, differentiating individuals with MCI from their healthy peers and labeling them as unsafe drivers [12,13,14]. Even subtle changes in cognitive functioning can influence driving performance, especially in demanding driving environments [10,15,16]. Issues such as misestimation of speed and distance from the leading car and maintenance of the correct lane have been noted as parameters that show deviations, with overall performance being significantly lower [12,15,16,17,18]. In contrast, a study by Economou et al. [19] found that patients with MCI reported driving at slower speeds and maintaining longer distances as safety measures while maintaining an average reaction time. This non-systematic finding highlights the need to distinguish between safe and unsafe drivers, with the latter having greater difficulty in conditions such as intersections, roundabouts, and parking [20].

Despite emerging deficits, individuals may remain able to drive safely through driving self-regulation, which depends on metacognitive awareness. Cognitive deficits are often accompanied by limited awareness, with a demonstrated link between misestimation of cognitive ability, driving self-awareness, and driving self-regulation [19,21]. Metacognition, or the knowledge of one’s cognitive system, facilitates awareness acquisition [22]. Since driving is considered a cognitive task, it is subject to metacognitive monitoring, which may lead to self-regulation. Metacognitive experiences, which are dynamic subjective experiences, manifest before, after, or during task processing and provide the basis for selecting regulatory processes when necessary [23]. These experiences include the metacognitive feeling of difficulty, estimation of effort, and feeling of certainty. The metacognitive feeling of difficulty refers to perceived interruptions in the flow of mental processing or existing problems in coordinating the mental actions required for a task. The metacognitive estimation of effort involves the perceived amount of effort necessary to complete a task. Similarly, the metacognitive feeling of certainty pertains to the perceived confidence in the correctness of a response, ranging from complete uncertainty to complete certainty [23]. Perceived self-efficacy, or the belief in one’s ability to organize and execute required actions, influences the formation of metacognitive experiences [24].

Drivers with MCI tend to overestimate their driving abilities, often referred to as illusory superiority, being up to 7.85 times more likely to overestimate their abilities [18] and consider themselves superior to the average driver [25,26]. This discrepancy between objective and subjective assessments of driving tasks is a stronger factor than objectively measured capacity and is negatively correlated with the likelihood of regulatory action, showing up to four times higher odds for unsafe driving [7,27,28,29]. Consequently, reduced driving self-awareness, along with perceived self-efficacy, significantly affects driving safety, especially when self-efficacy reaches unrealistic levels.

The extensive neuropsychological damage and progressive cerebral atrophy that characterize MCI may lead to inaccurate recognition of deficits, with the loss of awareness marking a transitional point from MCI to dementia [30]. The inability to metacognitively assess abilities decreases even from the initial stages, reaching a percentage up to 60%, with the individuals underreporting or even distorting their symptoms, highlighting a gap between metacognitive self-awareness and accurate self-evaluation [18,31]. A similar study found that 23% of drivers with MCI had reduced metacognitive awareness of their deficiencies [32]. These findings, along with a 0.6% annual decline in metacognitive proficiency [33], demonstrate age-related decreased awareness, emphasizing the importance of metacognitive awareness in daily activities [34,35].

Older drivers may not possess adequate metacognitive abilities to recognize the need for driving regulation, relying more on physical ability and feelings of discomfort rather than deliberate evaluation and metacognitive monitoring of driving parameters [36,37]. Regarding the effects of sex, women tend to report lower driving self-efficacy, with reported levels declining with age and health status [37,38]. Women seem to be more capable of recognizing changes in driving and tend to be more accurate in their self-reports, viewing driving as a mutable skill, in contrast to men, who see driving as a static skill [38,39]. Consequently, women may favor a metacognitive approach to their cognitive capabilities involved in driving, impacting the greater number of driving situations in which they self-regulate [40,41,42]. However, the greater discomfort and lesser need for driving may also influence women’s adoption of driving regulations rather than metacognitive awareness alone [43].

Thus, individuals may not recognize their inability to safely operate a vehicle, raising concerns about their physical integrity and that of others. This issue is supported by the lack of differences in driving cessation between individuals with MCI and their healthy peers, even when aware of their cognitive deterioration, attributed to reduced awareness of deficits and the consequent lack of need for adjustments [37,44]. Familiarity with the task can also hinder effective metacognitive monitoring and regulation, as older adults often avoid driving in certain circumstances due to an overall reduction in driving without necessarily increasing driving safety [37,45]. Effective regulation is contingent upon matching the individual’s mental potential [44,46]. However, self-regulation of driving is often perceived as an automated process requiring environmental cues for adjustment [26].

Self-awareness of deficits is related to the number and quality of compensatory strategies employed to compensate for weakened abilities [47]. Older drivers often adopt compensatory behaviors, with self-regulation increasing with age and perceived impairment [37,48]. Commonly avoided conditions include adverse weather, night driving, and unfamiliar areas, with such avoidance linked to lower driving skills [39,49,50,51]. Other regulated conditions include parking in demanding positions and driving during rush hours, on high-speed roads, or on slippery surfaces [47,48,52]. Using navigation data on 2131 older drivers, Molnar et al. [53] report similar compensatory strategies among men and women, with women maintaining greater awareness of their adoption.

In MCI drivers, a significant portion (62.4%, n = 362) reported adopting compensatory strategies in the past three months, such as avoiding night driving, driving in rain, challenging parking, and turning at highways [40]. Driving on busy roads as well as in unfamiliar areas was also found to be avoided more by MCI patients compared to their healthy peers [54]. In a Greek population survey (n =100), 77% of drivers with MCI reduced their driving distance, and 53% adopted lower speeds and avoided night driving [55]. However, many did not avoid driving in urban centers (66%), on highways (84%), without a passenger (88%), turning at difficult intersections (77%), or under time pressure (73%). These compensatory strategies were adopted less by healthy peers and more by dementia patients. Therefore, MCI appears to be a transitional stage between reduced strategy adoption and greater use, with driving self-regulation beginning as deficits emerge.

Schulz et al. [52] highlight that compensatory strategies may indicate unsafe driving, underscoring the need to investigate factors mediating their selection and implementation. Given that self-awareness of cognitive deficits relates to compensatory strategy quality and quantity, this study aims to investigate the metacognitive experiences of older adult drivers with MCI in specific driving scenarios. Relationships with demographics, perceived driving self-efficacy, and subjective cognitive function evaluations will be explored. The first hypothesis tested was that the demographic characteristics of sex, age, and education would predict the levels of metacognitive experiences reported by drivers with MCI. The metacognitive experiences included the metacognitive feeling of difficulty, the metacognitive feeling of certainty, and the metacognitive estimation of effort, which are products of metacognitive monitoring that provide the basis for subsequent regulation of the driving task. Women and older MCI patients were expected to report less metacognitive certainty and greater metacognitive difficulty and estimation of effort. No specific hypothesis was tested regarding the level of education.

The second hypothesis tested whether the subjective assessment of cognition, as measured through the cognitive difficulties an MCI patient acknowledges, and driving self-efficacy, referring to their belief in successfully executing driving, would mediate the aforementioned relationships. Therefore, metacognitive awareness and emerging metacognitive experiences in relation to specific driving conditions are expected to enhance our knowledge of the conscious adoption or non-adoption of compensatory strategies by older drivers with MCI.

## 2. Materials and Methods

### 2.1. Participants and Procedure

All participants enrolled in the study were MCI patients who had been neuropsychologically, neurologically, and via imaging methods and bio-indices examined and registered in the database of the Hellenic Association of Alzheimer’s Disease and Related Disorders (Alzheimer Hellas) during 2021–2022 (August 2021–August 2022). The inclusion criteria were: (a) an MCI diagnosis according to Petersen’s criteria [56,57] and (b) driving at least once a week at the time of the study. Exclusion criteria included the presence of depressive and/or psychiatric symptoms.

Consequently, 383 individuals who met the criteria were approached by telephone during July and August 2022. Of these, 110 did not respond to contact attempts, 15 refused, and 135 reported either not holding a driver’s license or having ceased driving. The total number of participants who voluntarily enrolled was 117, resulting in a response rate of 30.5%. Of these participants, 69 were women (57.3%) and 54 were men (42.7%), with a mean age of 72.06 years (SD = 5.49 years). Sixty-seven participants had a high-school education (medium level) (57.3%, 12 years), and 50 had a high level of education (42.7%, >12 years).

Participants were initially informed about the purpose of the study and the voluntary nature of their participation and were assured of their anonymity and confidentiality. After obtaining consent, the research tools detailed below were administered. Specifically, we administered driving scenarios accompanied by corresponding questions about their metacognitive experiences, the Cognitive Failures Questionnaire (CFQ) [58,59], and the Driving Self-Efficacy Scale (DSE) [60]. The average administration time was 30 min.

### 2.2. Ethics

All participants provided written informed consent during their initial clinical visit, agreeing that their basic demographic information, such as age, gender, and education, as well as their total scores on neuropsychological tests, could be used for research purposes. The study was approved by the Scientific and Ethics Committee of the Greek Association of Alzheimer’s Disease and Related Disorders (identifying number 90/08-06-2023). The Committee adheres to the General Data Protection Regulation (EU) 2016/679 of the European Parliament and the Council of 27 April 2016 on the protection of natural persons regarding the processing of personal data and the free movement of such data, as well as the principles outlined in the Helsinki Declaration.

### 2.3. Materials

#### 2.3.1. Metacognitive Experiences

Metacognitive experiences were examined through a set of nine driving scenarios formulated based on the statistically significant correlations that emerged from Katsouri’s doctoral thesis [55]. These scenarios targeted driving conditions that MCI drivers seem to self-regulate by applying compensatory strategies, such as driving in urban areas, driving during rush hour, driving on a highway, driving under time pressure, driving in unfamiliar areas, driving without a passenger, driving at difficult intersections, driving at night, and driving in the rain.

Each driving condition was examined within the context of a daily scenario. For example, the scenario for driving on a highway was presented as follows: “You have been informed that there is going to be a march in the city center, which may delay your arrival at your destination. Therefore, it would be better to travel via the ring road.” Each scenario was followed by three self-report questions related to the metacognitive experiences of the specific driving condition. These questions assessed the metacognitive feeling of difficulty (e.g., “How difficult do you find it to drive in these conditions?”), the metacognitive feeling of certainty (e.g., “How confident are you that you can do it?”), and the metacognitive appraisal of effort (e.g., “How much effort do you think you will need to exert?”). The questions remained constant and were repeated across the nine consecutive scenarios. Participants indicated their responses using a 4-point Likert scale (1 = not at all to 4 = high feeling).

#### 2.3.2. Subjective Evaluation of Cognition

The Cognitive Failures Questionnaire (CFQ) [58,59] was used as a subjective assessment of the participants’ cognitive difficulties. This 25-item self-report questionnaire examines the frequency of cognitive failures experienced in daily life over the past six months (e.g., “Do you ever not pay attention to traffic signs on the road?”). Participants indicated the frequency of cognitive errors using a 5-point Likert scale (1 = absence to 5 = high frequency). A higher total score indicates a greater extent of subjective cognitive failure.The Greek version of the questionnaire has been validated through exploratory factor analysis and confirmatory factor analysis. The results confirmed the tested one-factor model, with all loadings found to be statistically significant and positive. The instrument’s reliability is supported by a Cronbach’s α of 0.895, which is considered good [59].

#### 2.3.3. Driving Self-Efficacy

Driving self-efficacy was assessed using the Adelaide Driving Self-Efficacy Scale [60], a 12-item self-report questionnaire that examines drivers’ self-efficacy in 12 typical driving situations (e.g., “How confident do you feel driving in your area?”). Each question is rated on a 10-point Likert scale (0 = no self-efficacy to 10 = high self-efficacy). The final score is obtained by summing the responses, with a maximum score of 120 points; higher scores indicate higher perceived self-efficacy. The questionnaire was translated by the researcher and back-translated by an English teacher to ensure accuracy. It was then subjected to exploratory factor analysis. Principal component analysis with Varimax rotation revealed one factor that explained 68.50% of the total variance (KMO = 0.901, *p* < 0.001). The results confirmed the one-factor model, with all loadings being statistically significant and positive. The internal reliability was excellent, with a Cronbach’s α of 0.954.

### 2.4. Statistical Analyses

Mediation analysis is employed to understand whether a prognostic variable is related to an outcome variable, with this relationship potentially being influenced by other variables, known as mediators. This analysis computes both direct and indirect effects. Direct effects refer to the relationship between the prognostic and outcome variables, while indirect effects consider the influence of the mediators on this relationship. In this study, mediation analysis via formal path analysis was applied using JASP 0.18.03 to examine whether the demographic characteristics of MCI participants (age, gender, and education—prognostic variables) directly affect the formation of metacognitive experiences (outcome variables) related to specific driving scenarios. Additionally, the analysis explored whether this relationship is mediated by subjective evaluations of cognition and driving self-efficacy (potential mediators).

The analyses aimed to expand our understanding of whether the formation of metacognitive experiences is solely dependent on demographic characteristics (age, sex, and education) or is also influenced by the participants’ self-perceptions, as reflected in their driving self-efficacy and subjective cognitive evaluations. Given that metacognitive experiences underpin further regulatory actions and that individuals may have an altered self-image regarding their driving skills, it is crucial to clarify the potential mediating role of subjective measures in the relationship between demographic characteristics and metacognitive experiences. Therefore, mediation analysis is particularly suitable for this purpose.

## 3. Results

To examine the predictors of metacognitive experiences, including demographic characteristics, driving self-efficacy, and subjective cognition ratings, a mediation analysis was conducted using the statistical package JASP 0.18.03. The model tested included both direct effects of sex, age, and education, as well as indirect effects of these demographics through mean scores of the Cognitive Failures Questionnaire and the Driving Self-Efficacy Scale on the formation of three types of metacognitive experiences. The metacognitive experiences reported were entered into the model as outcome variables, represented by three mean scores: the mean score of feeling of difficulty, the mean score of feeling of certainty, and the mean score of estimation of effort. These mean scores were calculated by aggregating scores from each metacognitive experience reported in each of the nine driving scenarios and dividing by the number of scenarios. Table 1 presents the mean scores of each variable used, along with the mean scores of the metacognitive experiences in the nine driving scenarios. The overall results of the model tested are presented in Table 2 and Table 3 sequentially.

### 3.1. Mean Scores of the Variables Tested

As depicted in Table 1, the mean score for driving self-efficacy exhibited a notably high value, approaching the maximum score of 120 points. Specifically, among female participants, the mean score of driving self-efficacy was 100.06 (SD = 13.04), while among male participants, it was slightly higher at 106.30 (SD = 13.09). Conversely, the mean score of subjective evaluation of cognition appeared relatively low, suggesting a lesser extent of complaints related to cognitive functioning.

Concerning metacognitive experiences, a consistent pattern emerged across successive scenarios. Participants consistently reported lower levels of metacognitive difficulty and estimation of effort, juxtaposed with higher levels of metacognitive certainty. However, an exception was noted in the scenario involving driving in rain, where the reported metacognitive certainty was notably low. This deviation may be attributed to the heightened demands posed by this specific driving condition.

Overall, participants exhibited a tendency to perceive themselves as more capable of executing driving tasks while simultaneously displaying a reduced inclination to acknowledge potential difficulties associated with cognitive decline in daily living, particularly within the context of MCI. This pattern was also reflected in the formation of metacognitive experiences, where higher levels of certainty and lower levels of perceived difficulty and effort predominated across most driving scenarios.

### 3.2. Direct Effects of the Model Tested

Regarding the direct effects observed in the tested model (Table 2), a statistically significant direct effect of age on the mean score of the metacognitive feeling of certainty was identified. Notably, this effect was negative, indicating that as individuals advance in age, they exhibit decreased certainty regarding their ability to successfully perform driving tasks in demanding circumstances. Conversely, younger MCI drivers demonstrated a greater sense of certainty regarding the appropriate execution of driving tasks under discussion. These findings suggest that metacognitive feelings of certainty diminish with age, leading individuals to feel less reassured about their capability to navigate specific driving scenarios included in the study.

### 3.3. Indirect Effects of the Model Tested

In terms of the indirect effects observed in Table 3, a notable indirect effect of sex on the levels of metacognitive experiences was revealed through the driving self-efficacy reported by individuals. Specifically, for the metacognitive feeling of difficulty and the metacognitive estimation of effort, the effect was found to be negative, indicating that women, compared to men, tended to report lower driving self-efficacy, thus leading to a heightened perception of difficulty and effort required in navigating the driving scenarios examined. Conversely, for the metacognitive feeling of certainty, the indirect effect was positive. This suggests that women, due to their lower reported driving self-efficacy, exhibited a reduced sense of certainty regarding the appropriate execution of actions involved in specific driving conditions. In contrast, men, who reported higher levels of self-efficacy regarding their driving abilities, acknowledged a lesser degree of metacognitive difficulty and effort needed alongside a greater degree of certainty regarding their ability to navigate the driving circumstances involved. These findings underscore the influence of driving self-efficacy on the formation of metacognitive experiences, particularly highlighting the differential effects observed between genders.

## 4. Discussion

The primary objective of this study was to explore the level of metacognitive awareness among older adult drivers diagnosed with MCI, particularly within the context of specific driving conditions that demand heightened cognitive resources for safe execution. Through the examination of relative metacognitive experiences reported by participants, the study aimed to elucidate the interplay between these experiences and various factors, including demographic characteristics, subjective evaluation of cognition, and driving self-efficacy. Central inquiries revolved around understanding whether active older adult drivers with MCI possess insight into their capability for safe driving, as reflected in their metacognitive experiences. This insight was considered pivotal for the adoption of self-regulatory behaviors aimed at enhancing driving safety. Additionally, the study aimed to assess potential differences in metacognitive experiences between genders and across different age groups within the MCI. Women and older MCI people were expected to report lower metacognitive certainty and higher metacognitive difficulty and effort while the potential effects of education in these relationships were tested.

Regarding the first hypothesis, the data partially confirmed its premises. The study established a direct negative effect of age on the formation of the metacognitive feeling of certainty, indicating that older individuals diagnosed with MCI tend to exhibit less confidence in their ability to accomplish driving tasks under discussion. This finding aligns with existing literature, where age has been identified as a significant factor influencing decisions related to driving, with older age correlating with a higher likelihood of self-regulation or cessation of driving altogether [32,36,40,41,61]. The diminished certainty observed with increasing age may be attributed to a general decline in health and functional abilities typically associated with age [38,62]. As individuals perceive themselves as more vulnerable, their confidence in coordinating the actions necessary for successful driving diminishes. However, as underlined by Liang and his colleagues [36], feelings of frailty are often based on physical declines associated with aging rather than on cognitive declines, which are less easily self-monitored. This highlights the potential for inaccurate metacognitive judgments, particularly concerning driving skills. A decline in metacognitive reflection due to aging can lead to a false estimation of driving skills, especially concerning decrements in cognitive domains such as memory. Consequently, individuals may fail to recall driving errors accurately, leading to an overestimation of their driving proficiency and higher levels of the metacognitive feeling under discussion [44,47].

Regarding the second hypothesis of the study, the results provided only partial confirmation. Specifically, an indirect effect of sex on the formation of metacognitive experiences of older drivers with MCI through reported levels of driving self-efficacy was established. However, no significant effect of subjective cognitive failures in daily life was demonstrated. Women with MCI acknowledged lower metacognitive certainty and a higher degree of metacognitive difficulty and effort in the driving scenarios tested, largely due to their lower driving self-efficacy. This finding aligns with existing literature, which consistently shows that MCI women report lower levels of driving confidence and are more likely to recognize and admit a decline in their driving skills compared to men [57,63,64]. In this study, the mean levels of driving confidence reported by women were lower in relation to those reported by men, affecting and subsequently influencing the levels of metacognitive experiences.

Women appear more willing to accept changes in their driving skills, viewing these skills as adaptable. This perspective leads them to engage in more self-regulatory or avoidance behaviors in demanding driving situations. Conversely, men tend to view driving as a static skill and are more resistant to adopting regulatory behaviors [36,37,38,39,42,61]. Among MCI patients, older women drivers are particularly proactive, increasing the number of situations they regulate annually and having greater odds of engaging in driving self-regulation compared to men [45,61]. This heightened sensitivity and accuracy in detecting their own driving limitations [29] are reflected in the levels of driving confidence women report and the formation of the subsequent metacognitive experiences. This self-awareness serves as a crucial prerequisite for adjusting their driving behavior. Men, on the other hand, tend to be more confident in their driving skills and less likely to acknowledge potential declines and difficulties. This disparity may stem from traditional gender roles and instrumental attitudes associated with driving [42,43].

In addition to the direct and indirect effects already discussed, it is pertinent to briefly review the mean scores of the metacognitive experiences across the different driving scenarios. As reported in the Results section, a consistent pattern emerged: the metacognitive feeling of difficulty and estimation of effort were generally low, while the metacognitive feeling of certainty was higher. This pattern indicates a relative confidence among MCI patients in their driving abilities across various conditions. Driving at night or/and in rain were identified as the most challenging conditions for this group of drivers, as reflected in the elevated levels of metacognitive feeling of difficulty and effort reported and lower levels of metacognitive certainty. This finding aligns with the existing literature since they are the most usual circumstances that older drivers regulate through the implementation of compensatory strategies [40,47,49,50]. The high demands of these conditions likely diminish drivers’ certainty in their ability to perform safely. Interestingly, despite the recognized difficulty of driving at night, the mean score for the metacognitive feeling of certainty in this scenario was relatively high (mean score = 3.30 with a max score of 4 points). This suggests that older drivers may exhibit overly optimistic metacognitive judgments about their capabilities, potentially overlooking the cognitive and sensory challenges that nighttime driving entails. This self-favorable bias could pose significant safety risks, as it may lead to underestimation of the actual demands and overconfidence in their driving skills. Conversely, scenarios such as driving in urban areas, without another passenger, and/or on highways were perceived as less demanding. This perception is likely influenced by the participants’ familiarity with these conditions, as most were city dwellers accustomed to urban driving environments. The lower levels of reported metacognitive difficulty and effort, coupled with higher certainty in these scenarios, reflect a comfort derived from routine and experience.

The findings of this study underscore the critical role of realistic self-efficacy in influencing the metacognitive experiences of older drivers with MCI. Realistic self-efficacy, or the accurate assessment of one’s capabilities, directly affects metacognitive feelings and estimations, which, in turn, inform the behavioral control processes, such as determining the appropriate strategies to employ while driving. Despite the known cognitive declines associated with MCI, the participants in this study reported relatively high perceived driving self-efficacy, with an average score of 102.73 out of 120 points (SD = 13.37). This discrepancy between perceived and actual ability highlights a critical issue: the potential dissociation between self-efficacy and what individuals can realistically achieve. This overestimation of capabilities is particularly evident among male MCI drivers, who tend to maintain a high degree of driving self-efficacy despite cognitive impairments. The tendency to overestimate their cognitive and/or driving abilities can have significant implications for safety and self-regulation. High perceived driving self-efficacy, despite cognitive attenuation, may result in reduced perception of difficulty, less need to avoid challenging situations, decreased fear during driving, and a self-perception as safe drivers, perceptions that may hamper any possible regulative action [65]. During the administration of the driving scenarios, participants reported few modifications in their driving behavior. This finding is consistent with the literature, which suggests that MCI patients often do not adopt regulatory actions even when informed about their cognitive deficits [37], pointing out that this lack of behavioral modification persists and highlighting a critical area for intervention.

As highlighted by Baldock and his colleagues [63], driving self-efficacy and self-perception of driving ability are often more closely related to self-regulation than to actual driving skills, and self-efficacy beliefs have been shown to predict reductions in or avoidance of driving, even more strongly than objective measures of driving ability [66,67,68]. In the Multifactional Model of Enabling Driving Safety [46], actual driving behavior and risk-taking tendencies are distinguished from the drivers’ capacity. Self-monitoring and insight are highlighted as important moderators seen in this relationship, underlining the role of self-perceived ability as it is reflected in driving self-efficacy. Moreover, the MASRL model [69] elucidates the role of self-efficacy at the personal level. Self-efficacy influences top-down self-regulation, affecting the metacognitive experiences and the strategies implemented as a result. Consequently, the effects of driving self-efficacy on metacognitive experiences, or vice versa, since causal relationships could not be examined given the cross-sectional design of the study, are further understood.

The problem that we come across in this case, as mentioned before, is the accuracy of driving self-efficacy. Older drivers often fail to recognize and adapt to their limitations [70], and poorer self-awareness itself has been linked to functional and cognitive declines that MCI drivers may already face [71]. In some cases, poorer self-awareness has been linked not only to lesser driving self-regulation but also to a greater degree of ignorance of their involvement in situations potentially dangerous for themselves and others [72], highlighting the importance of being accurate. The tendency of older MCI drivers, especially men, to overestimate their abilities, as reported in the literature, can lead to self-evaluations that are favorable, helping to maintain a positive self-image despite cognitive decline [25,27,28]. However, this significantly impacts the formation of metacognitive experiences and the choices drivers make regarding driving circumstances and the effort they invest. This self-protective bias might explain why male participants in this study reported high levels of driving self-efficacy and low cognitive failures despite their MCI diagnosis. The biased beliefs they adopt, as shown by the perception of limited cognitive failures in their daily life, while simultaneously reporting high levels of self-efficacy, may function protectively for male people with MCI themselves, as noted by Freund and colleagues [27]. However, they raise the issue of effective self-regulation. As mentioned by Molnar et al. [29], the higher the individual’s perception of their abilities, the less likely they are to modify their driving behavior, which correlates with increased riskiness as a driver. The adoption of compensatory strategies, which increase linearly with perceived declines in specific driving parameters [37,47,48], further underscores the importance of accurate self-awareness.

## 5. Conclusions

To conclude, the formation of driving-related metacognitive experiences reported by older drivers with MCI appears to be influenced partially by age and indirectly by sex through driving self-efficacy. The lack of a significant effect from self-perceived cognitive failures, coupled with the high levels of driving self-efficacy reported at least by male MCI patients, suggests a reduced metacognitive awareness that characterizes older adult MCI drivers. This reduced awareness might lead to an overestimation of driving abilities, particularly among men, who maintain high confidence despite cognitive declines. Women, conversely, seem to have a greater acknowledgment of their limitations in driving, likely due to their lower self-efficacy.

Therefore, the need for accurate estimations of the strengths and limitations of older MCI drivers is pointed out. MCI does not imply unsafe driving; however, the person must adapt to the deterioration of cognitive functions that affect driving by applying compensatory strategies, which must be consciously chosen based on the person’s realistic perception. One way to achieve this is through shaping levels of self-efficacy in relation to what the individual can and cannot handle, which, as shown in the present study, influence the metacognitive experiences of the older driver with MCI and, consequently, the regulatory actions they will take.

Among the limitations of this study is the absence of any objective evaluation of driving to investigate the emerging metacognitive experiences more deeply. Subsequently, self-report instruments make it impossible to derive causal interpretations regarding the effects found. However, despite the existence of these limitations, which at the same time generate proposals for future study, the contribution of the present study lies in the importance of interventions that will orient the formation of realistic perception of abilities and weaknesses on behalf of older drivers diagnosed with MCI. Maintaining their status as a driver while realizing their real limits, which will lead them to undertake corresponding regulatory initiatives, emerges as the ultimate purpose of this study.

## Figures and Tables

**Table 1:** Mean score (and standard deviation) of the Cognitive Failures Questionnaire, the Driving Self-Efficacy Scale, and metacognitive experiences in driving scenarios.

		FOD	FOC	EOE
	Mean (SD)	Mean (SD)	Mean (SD)	Mean (SD)
Self-Efficacy	102.73 (13.37)			
CFQ	0.93 (0.52)			
FOD_MEAN	0.82 (0.87)			
FOC_MEAN	3.55 (0.56)			
EOE_MEAN	0.92 (0.90)			
Driving in Urban Areas		0.55 (1.12)	3.71 (0.72)	0.65 (1.26) 0.43
Driving at Rush Hour		0.79 (1.29)	3.59 (0.81)	0.65 (1.20)
Driving on a Highway		0.52 (1.12)	3.68 (0.78)	0.68 (1.20)
Driving Under Time Pressure	0.79	0.84 (1.22) 1.290	3.57 (7.69)	0.89 (1.25)
Driving in Unknown Areas		0.89 (1.38)	3.50 (0.89)	1.13 (1.39)
Driving Without a Passenger		0.43 (1.03)	3.74 (0.78)	0.44 (1.04)
Driving Difficult Intersections	0.52	0.86 (1.29) 1.126	3.56 (0.84)	0.91 (1.37) 1.35
Driving at Night		1.35 (1.45)	3.30 (1.02)	1.38 (1.44)
Driving in Rain		1.23 (1.41)	1.33 (0.94)	1.38 (1.41)

Note. CFQ = Cognitive Failures Questionnaire, FOD = feeling of difficulty, FOC = feeling of certainty, EOE = estimation of effort.

**Table 2:** The direct effects of age, sex, and educational level on three types of metacognitive experiences in MCI drivers.

Direct Effects								
							95% Confidence Interval	
			Estimate	Std. Error	z-Value	*p*	Lower	Upper
SEX	→	FOD_MEAN	−0.101	0.133	−0.755	0.450	−0.362	0.161
EDUC	→	FOD_MEAN	0.212	0.136	1.559	0.119	−0.055	0.479
AGE	→	FOD_MEAN	0.021	0.012	1.711	0.087	−0.003	0.045
SEX	→	FOC_MEAN	0.134	0.146	0.916	0.360	−0.152	0.420
EDUC	→	FOC_MEAN	−0.171	0.146	−1.172	0.241	−0.456	0.115
AGE	→	FOC_MEAN	−0.033	0.015	−2.234	0.025	−0.063	−0.004
SEX	→	EOE_MEAN	−0.174	0.140	−1.239	0.216	−0.449	0.101
EDUC	→	EOE_MEAN	0.168	0.133	1.262	0.207	−0.093	0.429
AGE	→	EOE_MEAN	0.023	0.014	1.685	0.092	−0.004	0.050

Note. Robust standard errors, robust confidence intervals, ML estimator. FOD = feeling of difficulty, FOC = feeling of certainty, EOE = estimation of effort.

**Table 3:** The indirect effects of age, sex, and educational level through subjective evaluation of cognition and driving self-efficacy on three types of metacognitive experiences in MCI drivers.

Indirect Effects										
									95% Confidence Interval	
					Estimate	Std. Error	z-Value	*p*	Lower	Upper
SEX	→	CF_MEAN	→	FOD_MEAN	−0.039	0.042	−0.922	0.357	−0.121	0.044
SEX	→	SUM_UP	→	FOD_MEAN	−0.318	0.121	−2.622	0.009	−0.556	−0.080
EDUC	→	CF_MEAN	→	FOD_MEAN	−0.022	0.040	−0.540	0.589	−0.101	0.057
EDUC	→	SUM_UP	→	FOD_MEAN	0.061	0.097	0.625	0.532	−0.130	0.251
AGE	→	CF_MEAN	→	FOD_MEAN	0.005	0.005	1.102	0.270	−0.004	0.014
AGE	→	SUM_UP	→	FOD_MEAN	0.016	0.011	1.476	0.140	−0.005	0.036
SEX	→	CF_MEAN	→	FOC_MEAN	0.029	0.032	0.903	0.366	−0.034	0.093
SEX	→	SUM_UP	→	FOC_MEAN	0.283	0.113	2.499	0.012	0.061	0.505
EDUC	→	CF_MEAN	→	FOC_MEAN	0.017	0.032	0.520	0.603	−0.046	0.079
EDUC	→	SUM_UP	→	FOC_MEAN	−0.054	0.087	−0.621	0.535	−0.224	0.116
AGE	→	CF_MEAN	→	FOC_MEAN	−0.004	0.004	−0.976	0.329	−0.011	0.004
AGE	→	SUM_UP	→	FOC_MEAN	−0.014	0.009	−1.501	0.133	−0.032	0.004
SEX	→	CF_MEAN	→	EOE_MEAN	−0.043	0.047	−0.911	0.362	−0.134	0.049
SEX	→	SUM_UP	→	EOE_MEAN	−0.307	0.120	−2.564	0.010	−0.541	−0.072
EDUC	→	CF_MEAN	→	EOE_MEAN	−0.024	0.045	−0.532	0.595	−0.113	0.065
EDUC	→	SUM_UP	→	EOE_MEAN	0.059	0.094	0.625	0.532	−0.125	0.242
AGE	→	CF_MEAN	→	EOE_MEAN	0.005	0.005	1.066	0.286	−0.005	0.016
AGE	→	SUM_UP	→	EOE_MEAN	0.015	0.010	1.456	0.145	−0.005	0.035

Note. Robust standard errors, robust confidence intervals, ML estimator. FOD = feeling of difficulty, FOC = feeling of certainty, EOE = estimation of effort.

## Data Availability

The raw data supporting the conclusions of this manuscript will be made available by the authors to any qualified researcher.
